# A Placebo-Controlled, Pseudo-Randomized, Crossover Trial of Botanical Agents for Gulf War Illness: Reishi Mushroom (*Ganoderma lucidum*), Stinging Nettle (*Urtica dioica*), and Epimedium (*Epimedium sagittatum*)

**DOI:** 10.3390/ijerph18073671

**Published:** 2021-04-01

**Authors:** Jarred Younger, Emily K. Donovan, Kathleen S. Hodgin, Timothy J. Ness

**Affiliations:** 1 Department of Psychology, University of Alabama at Birmingham, CH 233, 1300 University Boulevard, Birmingham, AL 35233, USA; kathleenhodgin@uabmc.edu; 2 Department of Psychology, Virginia Commonwealth University, White House, 806 West Franklin Street, Richmond, VA 23284, USA; donovanek@mymail.vcu.edu; 3 Department of Anesthesiology and Perioperative Medicine, University of Alabama at Birmingham, BMR2-208, 901 19th Street South, Birmingham, AL 35205, USA; tness@uabmc.edu

**Keywords:** reishi, stinging nettle, epimedium

## Abstract

This report is third in a three-part clinical trial series screening potential treatments for Gulf War Illness (GWI). The goal of the project was to rapidly identify agents to prioritize for further efficacy research. We used a placebo-controlled, pseudo-randomized, crossover design to test the effects of reishi mushroom (*Ganoderma lucidum*), stinging nettle (*Uritca dioica*), and epimedium (*Epimedium sagittatum*) in 29 men with GWI. Participants completed 30 days of symptom reports for baseline, then a botanical line consisting of 30 days of placebo, followed by 30 days each of lower-dose and higher-dose botanical. After completing a botanical line, participants were randomized to complete the protocol with another botanical, until they completed three botanical trials. GWI symptom severity, pain, and fatigue were contrasted between the four conditions (baseline, placebo, lower-dose, higher dose) using linear mixed models. GWI symptom severity was unchanged from placebo in the reishi lower-dose condition (*p* = 0.603), and was higher in the higher-dose condition (*p* = 0.012). Symptom severity was not decreased from placebo with lower-dose stinging nettle (*p* = 0.604), but was significantly decreased with higher-dose stinging nettle (*p* = 0.048). Epimedium showed no significant decreases of GWI symptoms in the lower (*p* = 0.936) or higher (*p* = 0.183) dose conditions. Stinging nettle, especially at higher daily dosages, may help reduce the symptoms of GWI. Epimedium does not appear to beneficially affect GWI symptom severity, and reishi may exaggerate symptoms in some GWI sufferers. These results are in a small sample and are preliminary. Further research is required to determine if stinging nettle is indeed helpful for the treatment of GWI, and what dosage is optimal. This trial was registered on ClinicalTrials.gov (NCT02909686).

## 1. Introduction

More than 30 years after the first description of Gulf War Illness (GWI), there is no scientific or medical consensus on the optimal approach for treatment [1]. As it is unclear whether a dedicated treatment will ever be available for GWI, promising existing medicinal agents should be explored for a role in GWI management.

This report is the third in a three-part clinical trial series investigating the effects of boswellia, curcumin, maritime pine bark, fisetin, luteolin, resveratrol, reishi, stinging nettle, and epimedium in Gulf War Illness. This report covers the results for reishi, stinging nettle, and epimedium. These agents were selected due to reported peripheral and central actions on inflammatory pathways. While abnormal inflammatory signaling is just one explanation for GWI, several lines of support for the hypothesis exist in the literature. Both animal model and human subject studies have identified abnormal inflammatory processes in the gut [2,3], brain [4,5] and general peripheral system [6,7].

Reishi (*Ganoderma lucidum*), also known as lingzhi, contains several triterpenoids [8,9] and polysaccharides [10,11] with demonstrated anti-inflammatory properties. Extracts such as lucidone D have shown analgesic properties in mice, associated with a decrease of nitric oxide, TNF-alpha and IL-6 expression [12]. The anti-inflammatory effects of reishi extracts appear to extend to microglia, downregulating the production of pro-inflammatory cytokines in lipopolysaccharide-stimulated cells [13], and promoting an M2 anti-inflammatory polarization [14]. Some preclinical trials have been reported in the literature; for example, a reduction of pro-inflammatory markers and clinical symptoms in a mouse model of systemic lupus erythematosus [15]. However, some reishi extracts have immunostimulating properties, promoting the production of IL-6, TNF-alpha, IL-17 and IFN-gamma [16,17] from macrophages and peripheral blood mononuclear cells. If different components of reishi can have opposing effects on the immune system, it is unclear how the whole mushroom will affect conditions of purported inflammatory origins. Clinical trials in inflammatory and autoimmune conditions are lacking, though one small trial in individuals with active rheumatoid arthritis [18] showed some evidence of efficacy.

Stinging nettle (*Urtica dioica*) has been investigated for rheumatoid arthritis, partially related to NF-kappa beta inhibition [19]. It is also studied in chronic colitis, with extracts reducing IL-1beta and TNF-alpha in a murine model [20]. Stinging nettle extracts are generally considered to be anti-inflammatory, and can reduce LPS-induced MCP-1 production and COX-2 expression in various cells [21] and TNF-alpha and IL-1beta in whole human blood [22]. Human research is limited, but a study in 50 men with diabetes type 2 showed nettle extract to reduce circulating TNF-alpha and C-reactive protein levels [23]. In healthy individuals, daily use of a leaf extract reduced whole blood, ex vivo, LPS-stimulated TNF-alpha and IL-1 beta production [24]. Results from studies are difficult to synthesize, given the wide variety of plant parts used, and the differing extraction methods. Some evidence supports the use of specific lipophilic dichloromethane extracts, rather than traditional (e.g., water or alcohol) extracts to increase the anti-inflammatory effects [25].

Epimedium (*Epimedium sagittatum*) is a flowering plant used in traditional Chinese medicine for pain, rheumatic and arthritis conditions. It contains the flavonoid icariin that has been studied for its suppressive effects on inflammatory pathways [26,27], including the reduction of microglia activation and neuronal loss in models of neuroinflammation [28]. It has been shown to reduce microglia activation and neurodegeneration in an LPS rat model of Parkinson’s Disease [29]. Its suppression of neuroinflammation has been proposed to be a result of upregulation of PPARγ that promotes M2 microglia polarization [30], inhibition of TAK1/IKK/NF-kappaB and JNK/p38 MAPK pathways [31], suppression of IRE1alpha-XBP1 pathways [32], and suppression of HMGB1-RAGE signaling [33].

In this study, we examined how the three botanical agents impacted GWI symptom severity, as well as pain and fatigue. Participants completed baseline and placebo conditions, followed by 30 days of lower-dose treatment and 30 days of higher-dose treatment. We hypothesized that the lower and higher dosages of the three botanical agents would decrease GWI symptom severity from both baseline and placebo.

## 2. Materials and Methods

This report is part of a larger clinical trial in which nine botanical agents were tested for effects in GWI. A pseudo-randomized, placebo-controlled, crossover design was used. Curcumin, boswellia, maritime pine, epimedium, fisetin, luteolin, reishi, resveratrol, and stinging nettle were tested in the larger study. In this paper, the results of reishi, stinging nettle, and epimedium are discussed. All procedures were approved by the Institutional Review Board of the University of Alabama at Birmingham (UAB: F150318011) in June 2015, and the trial was registered on ClinicalTrials.gov (NCT02909686) accessed on 21 September 2016.

### 2.1. Participants

Individuals screened for the study were men between the ages of 44 and 65 who met the Kansas GWI case criteria [34] and were deployed to the Persian Gulf region between 1990 and August 1991. As participants must have been at least 18 years old during the Persian Gulf War, the youngest possible age at the study start in 2016 was 44 years. All inclusion and exclusion criteria in the Kansas GWI criteria were utilized, with the exception that individuals with diabetes type 2 could participate. Individuals with diabetes could participate only if the disease was medically controlled, with hemoglobin A1C below 9%.

Individuals were excluded from participation if they were taking opioid analgesics, anti-inflammatory medications, nitroglycerine, or lithium, or if they reported allergies to any study compounds. Participants could not have blood/clotting disorders, hypotension (below 90/60 mmHg), cardiovascular disease, rheumatologic disorders, autoimmune conditions, or acute infection with a body temperature over 100.4°F. Individuals could not participate if their laboratory blood values showed an erythrocyte sedimentation rate greater than 40.0 mm/Hr, a C-reactive protein value greater than 10.0 mg/L, or positive rheumatoid factor or antinuclear antibody.

Individuals were also excluded if they scored 16 or greater on the Hospital Anxiety and Depression Scale (HADS; [35]) depressive subscale by summing the depression items rated on a 0 to 3 scale. Significant posttraumatic stress disorder (PTSD) was identified if individuals scored equal or greater than 50 on the 17-item PTSD Checklist—Military Version (PCL-M; [36]). Individuals with possible ongoing PTSD were subsequently assessed by a member of UAB’s Office of Psychiatric Clinical Research, using the Clinician Administered PTSD Scale (CAPS-5; [37]). The 30-item structured interview was used to make a final determination of current PTSD, using established criteria [38]. Individuals meeting criteria for current PTSD were excluded from the study.

### 2.2. Botanicals

Reishi was sourced from JHS Natural Products, Mushroom Science (Eugene, OR, USA) as a hot water and alcohol extract with 12% polysaccharides and 4% triterpene. Stinging nettle was procured from Nature’s Way (Green Bay, WI, USA) as pure nettle leaf. Epimedium was from Barlowe’s Herbal Elixirs (Palm Beach, FL, USA) as a 20% icariin extract. Procured agents were sent to a compounding pharmacy (Double Oak Mountain Pharmacy, Birmingham, AL, USA) for re-encapsulation. Materials were put in size 0 or 00 blue gelatin capsules and organized in weekly Cold Seal Compliance blister packs (Pharmacy Automation Supplies, Romeoville, IL, USA). Participants and research personnel were blinded to the botanical agent assignments.

Reishi was administered at 1600 mg (lower dosage) and 3200 mg (higher dosage) per day. Epimedium was given at 500 mg and 1000 mg per day (100 mg and 200 mg of icariin, respectively). Stinging nettle was given at 435 mg and 1305 mg per day. All botanicals were administered twice per day (morning and evening), with the total daily dosage being split evenly between the morning and evening doses. Placebo was administered in identical gelatin capsules with microcrystalline cellulose filler.

Individuals on any antihypertensive medications were not allowed to take reishi or epimedium, even if their blood pressure was well-controlled. Individuals were not assigned to take stinging nettle if they had any signs of diabetes, pre-diabetes, or were taking medications for diabetes. Individuals could participate in the study despite being excluded from taking one or more of the botanicals, as the entire list of nine botanicals had several contraindications. As many individuals with GWI also suffer from conditions such as high blood pressure and diabetes, excluding these individuals would have created a sample that is not representative of the general patient population.

### 2.3. Study Protocol

Individuals were screened via an online questionnaire and phone interview, followed by an in-person screening conducted at UAB’s Clinical Research Unit (CRU). Informed consent was obtained at the in-person visit, and blood samples were collected for further screening. As the screening involved blood tests, individuals provided informed consent before being determined eligible for participation. Therefore, several individuals who provided consent were not eligible to participate in the study. Consented individuals received a tablet device for completing daily symptom reports each evening for the duration of their study participation.

**Figure 1:**
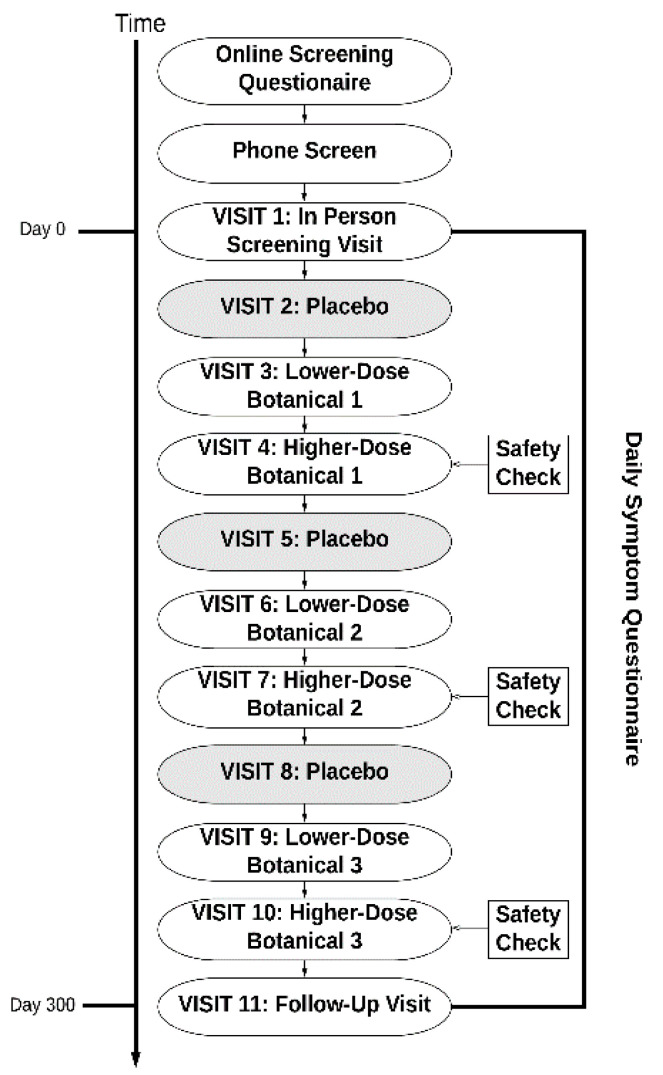
Study event flow. Participants took three of the tested compounds in the following order: placebo, lower-dose botanical, and higher-dose botanical.

Before beginning capsules, all participants completed a baseline period of 30 days. This period served as the baseline for all botanicals taken by the participant. Participants then were pseudo-randomized to receive up to three of the nine study compounds, as seen in Figure 1. A pseudo-randomized procedure was used to avoid contraindications with the botanicals. Randomization was conducted by a pharmacist. The pharmacist in charge of blinding and randomization was not otherwise involved in the study. The research personnel were blinded to the botanicals assigned to each participant. For each botanical, participants completed 30 days of placebo capsules, 30 days of lower-dose botanical, and 30 days of higher-dose botanical. Participants were blinded to all aspects of the study (the protocol and botanicals assigned). Research personnel were blinded to the botanicals assigned, though they were aware that placebo occurred first, followed by lower-dose and higher-dose treatment.

Throughout their involvement with the study, participants returned to the CRU every month. At these visits, participants would obtain a new supply of study capsules. Blood samples were also obtained at visits 4, 7, and 10 to conduct safety tests. Standard clinical renal and hepatic panels were performed. Participants first took the lower dosage and then the higher dosage of the botanical so that any renal or hepatic issues could be detected before the participant took the larger dosage. Participants received compensation during each visit. The participants received a total of $1500 for attending all 11 of the study visits and completing the protocol. Participants who successfully completed all three botanical assignments were allowed to enroll in the study a second time, where they would be assigned new botanicals.

### 2.4. Outcome Measures

Participants completed self-reported symptom severity measures every evening during the study, using the Qualtrics Research Suite (Qualtrics, Provo, UT, USA) on a tablet. The primary outcome used in this clinical trial was a 0–100 item assessing overall GWI severity. The item read, “Overall, how severe have your symptoms been today?” The zero response was anchored by “No symptoms at all” and 100 was labelled as “Severe symptoms”. This generic symptom response was chosen because GWI sufferers can have a wide range of principal complaints.

### 2.5. Secondary Outcome Measures

To further explore the impact of botanicals on common GWI complaints, we also measured changes in pain and fatigue severity. Both outcomes were measured on a 0–100 scale. Pain was measured by the item, “Overall how severe is your pain?” from “No pain at all” to “Severe pain”. Fatigue was measured by “How fatigued have you felt today?” from “Not fatigued at all” to “Severely fatigued”. Other GWI symptom domains such as respiratory and skin issues were rarely endorsed by participants and were not analyzed.

### 2.6. Statistical Analyses

All analyses were conducted in SPSS version 24 (IBM Corp., Armonk, NY, USA). For each botanical, a linear mixed model (LMM) was created to test changes in the primary outcome. LMMs were used to accommodate the repeated outcome assessments in each condition, nested in each participant. Subject ID was the repeated-measures nesting variable, the repeated-measures index was the day in the study, and the repeated measures covariance type was compound symmetry. The predictor was study condition, which could take four values (baseline, placebo, lower-dose, and higher dose botanical). The last 14 days of each condition were included in the models. The final 14 days of the baseline condition at the beginning of participation were used as the baseline for all tested botanicals. Post hoc tests were conducted using least-squares differences. A *p* < 0.05 was used for all tests. The same statistical approach was used to test the secondary outcomes of pain and fatigue. Nine total models are presented in this report (three botanicals tested for the outcomes of GWI severity, pain and fatigue).

If a participant did not complete the protocol, any valid data were still used in statistical tests. However, data imputation of missing values was not used, and participants were not included if they never took the assigned botanical. Therefore, we did not use a complete intent-to-treat method for analysis.

The three trials were powered to detect within-person fixed effects for study condition (baseline, placebo, lower dosage and higher dosage). Detection of a medium effect size (Cohen’s d = 0.5) was targeted at a threshold of *p* < 0.05. With 56 repeated outcome assessments per participant, and a repeated measures correlation of 0.5, it would require 10 individuals to achieve 0.99 power for the main effect test.

## 3. Results

### 3.1. Participant Attrition 

**Figure 2:**
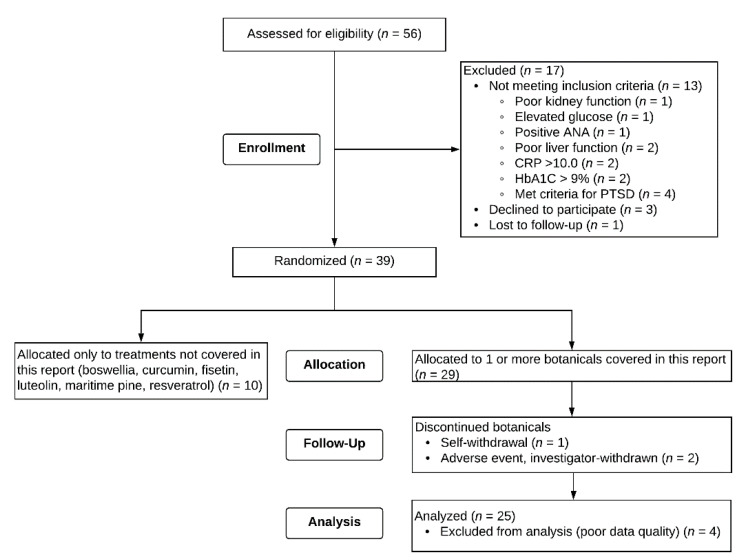
CONSORT Flow diagram. The three participants who discontinued botanicals provided sufficient data to be included in analyses. Four participants could not be included in analyses due to unusable outcome responses (two reported only “zeros” for GWI severity, and two gave all retrospective reports).

**Figure 3:**
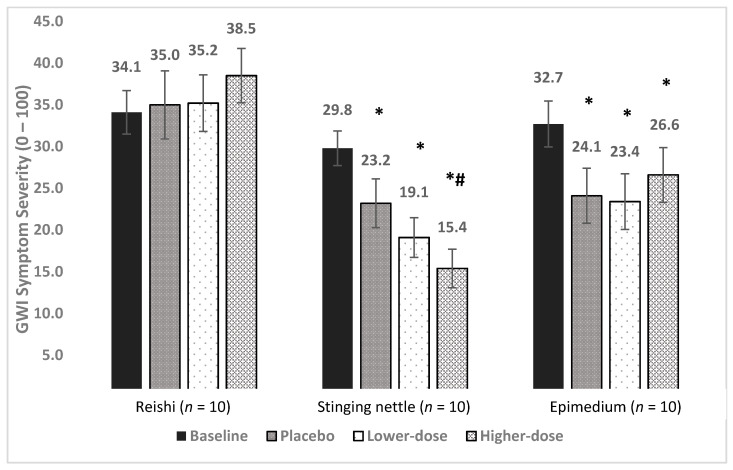
Main treatment effects of reishi, stinging nettle, and epimedium on GWI symptom severity. Average symptom levels (0–100) are presented for the baseline, placebo, lower-dose, and higher-dose conditions. * = significantly lower than baseline, *# = significantly lower than baseline and placebo (*p*’s < 0.05).

In the larger study, 56 men gave consent for participation, and 39 of those individuals were eligible and started study procedures. Twenty-nine of those individuals received at least one of the agents tested in this report. Data from four participants were excluded due to poor quality. Two of the individuals rated their GWI symptom severity as zero throughout the protocol, with no variability. Two other individuals gave all retrospective reports which were not considered valid. Data from the remaining 25 participants were analyzed, which includes data from three participants who discontinued botanicals for reasons indicated in Figure 2. The ages were 46 to 65 (M = 51.36, SD = 4.42). Nineteen (76%) of the participants identified as non-Hispanic White, five (20%) as non-Hispanic Black, and one (4%) as Hispanic. Of the twenty-five participants, one individual self-withdrew during the study and one individual was investigator-withdrawn due to adverse events. Data from these individuals who partially completed the study were still included in analyses (Figure 3).

Five participants were randomly assigned two of the botanicals in this report. All other participants took only one of the botanicals in this report (no individuals received all three botanicals discussed here).

### 3.2. Reishi

Ten individuals completed the protocol. Average age, body mass index (BMI), systolic blood pressure (SBP), diastolic blood pressure (DBP), heart rate (HR), and body temperature (temp) were collected. Blood values for glucose, erythrocyte sedimentation rate (ESR), and C-reactive protein (CRP) were provided.

The average age was 51.1 years (SD = 3.0), BMI was 32.4 (SD = 6.5), SBP was 138.1 mmHg (SD = 10.4), DBP was 84.0 mmHg (SD = 7.9), HR was 72.2 (SD = 7.7) and body temperature was 98.1°F (SD = 0.4). Glucose was 112.7 mg/dL (SD = 35.3), ESR was 9.3 mm/Hr (SD = 7.9) and CRP was 3.3 mg/L (SD = 2.2).

Baseline symptom severity was 34.1. Symptom severity was 35.0 in placebo, 35.2 in lower-dose reishi, and 38.5 in higher-dose reishi, representing a 2.6%, 3.2%, and 12.9% increase, respectively (Figure 2).

The LMM showed a significant main effect for condition [F (3, 615) = 11.2, *p* < 0.0001]. Post hoc, pairwise contrasts showed symptom severity to be higher in all conditions versus baseline: placebo (*p* = 0.005), lower-dose (*p* = 0.001), and higher-dose (*p* < 0.0001). There was no significant difference between placebo and lower-dose reishi (*p* = 0.603). Symptom severity was greater in higher-dose reishi than in both placebo (*p* = 0.012) and lower-dose reishi (*p* = 0.046). Reishi was associated with an increase of GWI symptom severity.

Reishi was the first treatment taken by five participants, the second treatment for two participants, the third treatment taken by one participant, and the fourth for two participants. We also examined what was taken by these 10 individuals before reishi. Two of the participants took curcumin before reishi, two took epimedium, and one took fisetin before reishi.

### 3.3. Stinging Nettle

Ten individuals were included in analyses. Two individuals were removed from the study because of adverse events (one had elevated creatine kinase and one had increasing headache severity), but they both provided sufficient data for analyses. These two individuals did not have data available for the higher-dosage condition. The average age was 51.4 years (SD = 5.4), BMI was 33.6 (SD = 4.4), SBP was 143.1 mm/Hg (SD = 11.4), DBP was 86.9 mm/Hg (SD = 6.0), HR was 67.8 (SD = 10.7) and body temperature was 98.4°F (SD = 0.3). Glucose was 103.9 mg/dL (SD = 15.7), ESR was 4.1 mm/Hr (SD = 3.8) and CRP was 4.5 mg/L (SD = 2.2).

Baseline overall symptom severity was 29.8. Symptom severity was 23.2 during placebo, 19.1 during lower-dose nettle, and 15.4 in higher-dose nettle, representing a 22.1%, 35.9%, and 48.3% drop, respectively (Figure 2).

The LMM showed a significant main effect for condition [F (3, 626) = 28.55, *p* < 0.0001]. Post hoc pairwise contrasts showed that symptom severity was reduced significantly from baseline in all conditions (*p*’s < 0.0001). Lower-dose nettle was not significantly different from placebo (*p* = 0.604). Higher-dose nettle was significantly different from placebo (*p* = 0.048), but not lower-dose nettle (*p* = 0.142).

Stinging nettle was the first treatment received for five participants, second for two participants, third for two participants, and fifth for one participant. Three received pine bark before stinging nettle. One received reishi mushroom, and one received boswellia before starting stinging nettle.

### 3.4. Epimedium

Ten individuals completed the protocol. The average age was 50.3 years (SD = 3.7), BMI was 31.2 (SD = 4.4), SBP was 132.4 mm/Hg (SD = 12.0), DBP was 85.8 mm/Hg (SD = 6.2), HR was 74.6 (SD = 8.9), and body temperature was 98.2°F (SD = 0.4). Glucose was 99.4 mg/dL (SD = 13.3), ESR was 6.1 mm/Hr (SD = 5.3) and CRP was 4.9 mg/L (SD = 3.2).

Baseline symptom severity was 32.7. Symptom severity was 24.1 in placebo, 23.4 in lower-dose epimedium, and 26.6 in higher-dose epimedium, representing a 26.3%, 28.4%, and 18.7% reduction in severity, respectively. The LMM showed a significant effect for condition [F (3, 635) = 13.21, *p* < 0.0001]. Post hoc, pairwise contrasts showed that severity was lower in all conditions than in baseline (*p* < 0.0001, *p* < 0.0001, and *p* = 0.0004). Neither lower-dose (*p* = 0.936) nor higher-dose (*p* = 0.183) epimedium differed from placebo. There was also no difference between lower-dose and higher-dose epimedium (*p* = 0.224). No clinical benefits were observed for epimedium.

For two individuals, epimedium was the first treatment given. For five individuals, it was the second treatment they received. For three individuals, it was the third treatment received. Four individuals received luteolin before epimedium, three received resveratrol and one received reishi.

### 3.5. Secondary Outcomes

Table 1 shows average pain and fatigue in each of the conditions. No significant differences from placebo were observed for any of the tested compounds (reishi, stinging nettle, or epimedium).

### 3.6. Adverse Events

Adverse events can be found in Table 2. Diarrhea was reported for each of the tested compounds and was the most likely event to be reported in the placebo condition.

## 4. Discussion

In this study, reishi, stinging nettle, and epimedium were screened as potential treatments for GWI. No clinical benefits were observed for daily use of reishi or epimedium. However, stinging nettle was associated with a decrease in GWI symptom severity. The treatments were well-tolerated.

Of the three botanicals studied in this report, only stinging nettle showed clinical benefits in GWI patients. Symptom severity was reduced by 35.9% with the lower-dose administration, and by 48.3% in the higher-dose administration. Pain and fatigue were also lowest in the higher-dose condition, though no significant differences from placebo were observed. We are not aware of any other clinical trials specifically testing oral stinging nettle for treating autoimmune, inflammatory, or pain disorders; however, several published trials have examined topical preparations for chronic musculoskeletal pain [39,40,41]. Additionally, stinging nettle has been tested in proprietary oral formulas with other agents for treating osteoarthritis pain [42]. Preliminarily, we see that oral stinging nettle treatment at the 1305 mg per day dosage may be helpful for GWI. However, potential negative effects were also observed for this botanical, with one individual experiencing increased creatine kinase and another reporting greater headache severity.

An unexpected effect was observed in the reishi treatment—a significant increase of GWI severity in the lower- and higher-dose conditions. While most extracted components of reishi are reported to have anti-inflammatory properties, it is possible that the whole reishi mushroom, given at these dosages, enhance inflammatory processes. However, it is notable that the ten individuals receiving the treatment did not show the typical 20–25% drop of GWI symptom severity in the placebo condition. Rather, severity went up negligibly during placebo. There were no anomalies in the data of any individuals and no reason why expectations would be different in this group than in any other group studied. We also examined the consistency of self-report symptom severity during placebo as a basic test of order effects. Participants taking reishi gave similar severity scores each time they started a new placebo condition (Cronbach’s alpha = 0.96). This internal consistency was also seen in those taking stinging nettle (0.95) and epimedium (0.91). Given the atypical placebo effect, we are hesitant to conclude that reishi can exacerbate GWI symptoms, but it must be considered. We determined that reishi, at 1600 mg and 3200 mg per day is not likely to be helpful for managing GWI.

Epimedium did not show any clinical benefit for GWI symptom severity. Participants showed a typical placebo response, with no additional drop of symptom severity in the lower or higher dosage conditions. We cannot contrast these results with other clinical trials, as there are no published trials of epimedium for chronic pain or inflammation. We determine that epimedium at 1000 mg or 2000 mg per day was not helpful for GWI symptom severity.

While we have focused on anti-inflammatory effects of these botanicals, all three have demonstrated effects on other systems. Reishi has been widely investigated for its antioxidative and free radical scavenging properties [43]. Antitumor, antimicrobial, and acetylcholinesterase effects have also been demonstrated [44]. Stinging nettle similarly has multiple observed effects apart from anti-inflammatory actions. For example, beneficial effects on hypertension and diabetes have been reported [45]. Epimedium also has a wide range of pharmacological effects, with several papers reporting antioxidative, neuroprotective, and several other clinically beneficial effects [46]. As each of the agents tested in this study have several effects potentially useful in GWI, it is difficult to determine which individuals may respond to treatment and why those individuals improve. We cannot conclusively attribute beneficial effects of stinging nettle on GWI to anti-inflammatory effects. Future clinical testing of promising botanicals should test inflammation and other blood markers to identify mechanisms of clinical benefit.

### Limitations

This study was designed to identify substances to prioritize for further study in GWI treatment. Due to the small sample size, no treatment recommendations can be made based on the reported findings. While this study allowed several potential treatments to be rapidly screened, drawbacks to the process exist. Generalizability to the larger GWI population is limited, as only men were included. Baseline GWI severity, as well as baseline pain and fatigue, was generally mild in these groups (around 30 out of 100 severity). It is unknown if the tested agents would have stronger or weaker effects on severe GWI. The small sample size also precludes statistical evaluation of adverse side-effects, and it is not possible to estimate what side-effects would occur in a larger population.

We used a single-item measure of GWI severity because no single symptom is shared by all individuals with the illness. Outcome assessments were completed daily, and longer GWI measurement tools may have been too burdensome for daily use. The item used as the primary outcome has not been validated, and it may not accurately capture changes in GWI severity. Future work should use standard outcome assessments.

This study design also kept participants on each substance and dosage for a limited time (one month). The short treatment period could miss clinical benefits that take longer to develop. The short treatment also means that the long-term benefits of the tested substances are unknown. We were also able to test only two dosage levels for each compound. While the best practices in botanical treatment were used in selecting the dosages, we do not know if lower or higher dosages would have been even more effective. Further, the design always placed the lower dosage before the higher dosage of each botanical so safety tests (renal and hepatic) could be conducted. By having lower dosage before higher, the effect of dosage cannot be separated from the effect of treatment duration. Individuals improving in the higher dosage condition may simply have benefited from taking the agent for a longer period of time.

A significant aspect of the design is the short time between switching from one botanical to the next. After ending one botanical, participants directly started the next trial. There were only two weeks before participants reached the placebo period for statistical analysis. Two weeks may have been insufficient for individuals to return to their baseline levels of symptom severity. Positive or negative responses to the previous botanical could bleed over and have an impact on the next trial. Order effects could be present, though we are unable to statistically test for them because of the small sample size and hundreds of possible treatment orders. Future studies testing multiple potential treatments should include longer washout periods between trials.

An additional caveat with botanical treatment research is that products can vary significantly in purity, ratios of active components, presence of added compounds to enhance effects, quality of packaging, and many other factors. Individuals may have different experiences when testing commercially available botanicals, given that many companies provide the products tested in this report.

## 5. Conclusions

Of the three compounds discussed in this report, stinging nettle was the only one to show benefit in GWI. Stinging nettle may therefore be prioritized for larger testing in GWI. It is critical to managing GWI that the more promising therapeutic compounds be rapidly identified, as the window to make a meaningful impact diminishes with time. As botanicals are already available to the general population, the time from successful clinical trials to use by patients is shorter than with new pharmaceuticals. Therefore, we hope that several groups will prioritize and test new potential GWI treatments.

## Figures and Tables

**Table 1:** Means (and standard deviations) and linear mixed model (LMM) results for the secondary outcomes of self-reported pain and fatigue. Results are presented separately for reishi, stinging nettle, and epimedium.

	Baseline	Placebo	Lower Dose	Higher Dose	LMM
			Reishi		
Pain	31.1 (24.0)	31.9 (23.5)	32.2 (24.7)	33.8 (25.6)	F (3, 549) = 2.6, *p* = 0.050
Fatigue	31.2 (21.8)	28.6 (19.4)	30.4 (17.8)	31.9 (17.9)	F (3, 556) = 5.5, *p* = 0.001
Stinging Nettle					
Pain	29.3 (16.4)	22.5 (21.4) *	18.2 (14.6) *	17.8 (13.2) *	F (3, 626) = 26.1, *p* < 0.0001
Fatigue	40.6 (23.1)	40.3 (27.6)	38.6 (26.7)	33.5 (27.4)	F (3, 626) = 0.93, *p* = 0.424
Epimedium					
Pain	29.4 (20.7)	23.4 (14.8) *	24.4 (19.5) *	24.3 (16.8) *	F (3, 635) = 8.4, *p* < 0.0001
Fatigue	33.2 (20.8)	32.2 (19.8)	30.4 (19.5) *	34.7 (21.7)	F (3, 635) = 3.8, *p* = 0.010

* = significantly lower than baseline.

**Table 2:** Incidence of self-reported adverse events.

	Reishi			Stinging Nettle			Epimedium		
Adverse Event	P	LD	HD	P	LD	HD	P	LD	HD
Diarrhea	-	2	1	1	1	-	1	2	-
Elevated creatinine kinase	-	-	-	-	1	-	-	1	-
Flushing	-	1	1	-	-	-	-	-	-
Headaches	-	1	-	2	-	-	-	-	-
Migraine	2	-	1	-	-	-	-	-	-
Upset stomach, lower GI	1	1	1	-	-	-	-	-	-
Worsening fatigue	1	1	1	-	-	-	-	-	-
Worsening GERD	1	1	1	-	-	-	-	-	-

P = placebo, LD = lower dose, HD = higher dose, GERD = gastroesophageal reflux disease, GI: gastrointestinal.

## Data Availability

The datasets used and/or analyzed during the current study are available from the corresponding author upon reasonable request and the approval of the data owner.
